# Polymorphisms in bovine immune genes and their associations with somatic cell count and milk production in dairy cattle

**DOI:** 10.1186/1471-2156-11-99

**Published:** 2010-11-05

**Authors:** Christine Beecher, Mairead Daly, Stuart Childs, Donagh P Berry, David A Magee, Tommie V McCarthy, Linda Giblin

**Affiliations:** 1 Teagasc Food Research Centre, Moorepark, Fermoy, Co. Cork, Ireland; 2 Biochemistry Department, University College Cork, Cork, Ireland; 3 Teagasc, Animal and Bioscience Research Department, Animal and Grassland Research and Innovation Centre, Moorepark, Fermoy, Co. Cork, Ireland; 4 Animal Genomics Laboratory, UCD School of Agriculture, Food Science and Veterinary Medicine, University College Dublin, Belfield, Dublin 4, Ireland

## Abstract

**Background:**

Mastitis, an inflammation of the mammary gland, is a major source of economic loss on dairy farms. The aim of this study was to quantify the associations between two previously identified polymorphisms in the bovine toll-like receptor 2 (*TLR2*) and chemokine receptor 1 (*CXCR1*) genes and mammary health indictor traits in (a) 246 lactating dairy cow contemporaries representing five breeds from one research farm and (b) 848 Holstein-Friesian bulls that represent a large proportion of the Irish dairy germplasm. To expand the study, a further 14 polymorphisms in immune genes were included for association studies in the bull population.

**Results:**

TLR4-2021 associated (P < 0.05) with both milk protein and fat percentage in late lactation (P < 0.01) within the cow cohort. No association was observed between this polymorphism and either yield or composition of milk within the bull population. CXCR1-777 significantly associated (P < 0.05) with fat yield in the bull population and tended to associate (P < 0.1) with somatic cell score (SCS) in the cows genotyped. CD14-1908 A allele was found to associate with increased (P < 0.05) milk fat and protein yield and also tended to associate with increased (P < 0.1) milk yield. A *SERPINA1 *haplotype with superior genetic merit for milk protein yield and milk fat percentage (P < 0.05) was also identified.

**Conclusion:**

Of the sixteen polymorphisms in seven immune genes genotyped, just CXCR1-777 tended to associate with SCS, albeit only in the on-farm study. The lack of an association between the polymorphisms with SCS in the Holstein-Friesian data set would question the potential importance of these variants in selection for improved mastitis resistance in the Holstein-Friesian cow.

## Background

Mastitis, an inflammation of the mammary gland, is a major source of economic loss on dairy farms. The disease cost to the EU dairy industry was €1.5 billion in 2005 [1]. This is due to increased involuntary culling [2], therapeutic costs [3], reduced milk yield and changes in milk composition [4]. Although farm management practices play a major role in mastitis incidence and control [5], modern day breeding programmes aim to incorporate increased mastitis resistance in the dairy herd without compromising milk production [6]. With the exception of the Nordic countries [7], most countries are limited to select for animals with low somatic cell count (SCC) due to the lack of routinely available phenotypic data on clinical mastitis. Indeed it has been shown in sheep that animals bred for low somatic cell score (SCS; natural logarithm of SCC) possess a better ability to limit and eliminate mastitis infections than their high SCS counterparts [8]. Mastitis resistance/susceptibility is a complex multi-locus trait and unsurprisingly, QTL for mastitis and/or SCC have been identified on almost all chromosomes of the bovine genome [9]. With the development of high density single nucleotide polymorphism (SNP) chips, genome wide association studies (GWAS) are becoming popular for breeding programmes. While GWAS has advantages, large studies are needed to find significance after adjusting for multiple testing [10,11]. Furthermore, fine mapping is still needed to efficiently identify the candidate gene and/or causative mutation [11].

Given that the host response to mastitis is complex and involves many different genes and cellular pathways [12], genes coding for immune factors that detect and eliminate pathogens are strong potential markers. For example, Toll-like Receptor 4 (TLR4) is an innate immune protein on cell surfaces that recognizes lipopolysaccharide (LPS) of Gram-negative bacteria [13]. The TLR4-2021 (C/T) polymorphism, within the third exon, results in the substitution of threonine with isoleucine at amino acid position 674, located within the transmembrane-cytoplasmic domain [14]. The C allele associates with decreased SCS and higher lactation persistency in the daughters of Holstein-Friesian bulls [15]. Combined with this, *TLR4 *exhibits a differential expression during mastitis episodes [16,17] which prompted Ogorevc et al. [9] to propose *TLR4 *as a strong candidate for inclusion in cattle breeding programs to augment the accuracy of selection for mastitis resistance.

*CXCR1 *encodes a chemokine receptor on neutrophil surfaces which binds the proinflammatory cytokine interleukin 8 (IL8) with high affinity. The bovine *CXCR1 *gene maps to a position of 110,617,059,110 megabase-pairs (Mbp) on chromosome (BTA) 2 and codes for a protein of 360 amino acids in length. The CXCR1-777 (G/C) single nucleotide polymorphism (SNP) results in a glutamine to histidine substitution at amino acid position 245 located within the third intracellular loop of CXCR1 [18]. When CXCR1 is stimulated by IL8, this loop is implicated in G-protein binding and calcium signaling [19]. The CC genotype of CXCR1-777 associates with impaired neutrophil migration, impaired reactive oxygen species production *in vitro *and increased subclinical mastitis *in vivo *[18,20].

Other potential immune targets for incorporation into breeding programs include polymorphisms in the cluster of differentiation 14 (*CD14*), caspase recruitment domain 15 (*CARD15*) and *IL8 *genes. *CD14 *encodes a membrane-associated protein on the surface of cells which is involved in the recognition of LPS in conjunction with TLR4 [21]. Of the exonic SNPs identified in the *CD14 *gene, CD14-1908 (A/G) causes an asparagine to aspartic acid substitution at amino acid position 175 within the leucine rich repeat (LRR) domain involved in pathogen recognition [22]. Animals with the mutant variant have a higher percentage of neutrophils expressing CD14 molecules on their surface which may increase the speed of response to pathogen attack [22]. CARD15 is a cytosolic protein that initiates inflammation following microbe recognition [23] and plays an important role in nuclear factor-kappa B (NF-κB) activation [24]. The CARD15-3168 (A/T) polymorphism, within exon 12, results in a leucine_125 _to glutamine switch within the LRR domain and is associated with increased SCS values [25]. IL8 is a potent inducer of chemotaxis and attracts and activates neutrophils in the early stages of host defense against bacterial invasion [26]. Several IL8 SNPs have been identified [27] and associations with milk fat yield and udder depth have been reported [28].

*SERPINA1 *encodes a serum protease inhibitor, whose primary role is to protect tissues against neutrophil attack. In particular, secretion of this protease protects 'self' cells from neutrophil elastase digestion and may play a role in the maintenance of milk quality over the lactation curve [29]. Indeed a *SERPINA1 *haplotype, composed of five SNPs within exon regions (SERPINA1-164, -269, -284, -407, -989), is associated with decreased SCS, increased milk yield, milk fat yield and productive life [30].

The main objective of this study was to quantify the associations between CXCR1-777 and TLR4-2021 with milk performance and somatic cell traits using both individual cow production data and estimated genetic merit of Holstein-Friesian sires based on progeny performance. The advantage of an experimental design that uses direct phenotypic data from individual cows is that it allows for the inclusion of deeper phenotypes and various dairy breeds but the results are only applicable to (a) the family lines and (b) farm management practices and environments similar to those described. In comparison, the performance traits evaluated in the Holstein-Friesian sires are not expressed by the sires themselves. The advantage of this alternative design is the increased study power, and therefore increased accuracy, to detect associations by minimizing the proportion of variance in the trait under investigation attributable to non-genetic variation. The disadvantage is that performance traits included in the analyses are limited to traits routinely measured on progeny.

## Results

### Dairy cows

Genotype frequencies of TLR4-2021 and CXCR1-777 and summary statistics in the on-farm study are summarized in Tables 1, 2 and 3. For TLR4-2021, Jersey and Jersey x Holstein-Friesian crossbreds had a lower proportion of CC homozygotes, than their herdmates. The rare TT genotype was absent in the Montbeliarde and Norwegian Red breeds. For CXCR1-777, heterozygous CG was the most abundant genotype, within breed, with the exception of the Jersey cows where the GG genotype predominated (62%).

**Table 1:** Allele frequencies of CXCR1-777 and TLR4-2021 in dairy cow cohort

SNP^1^	Genbank	Genotype	Entire Herd *n *= 246 (%)	Holstein-Friesian *n *= 77 (%)	Jersey *n *= 47 (%)	Holstein-Friesian x Jersey Cross *n *= 47 (%)	Montbeliarde *n *= 24 (%)	Norwegian Red *n *= 51 (%)
TLR4-2021	NM_174198	T/T	0.1	0.03	0.29	0.22	0	0
		T/C	0.56	0.46	0.62	0.73	0.37	0.61
		C/C	0.34	0.51	0.09	0.05	0.63	0.39
								
CXCR1-777	U19947	C/C	0.2	0.14	0.13	0.17	0.375	0.28
		C/G	0.42	0.5	0.25	0.46	0.375	0.47
		G/G	0.38	0.36	0.62	0.37	0.25	0.25

^1 ^SNPs genotyped by PCR-RFLP

**Table 2:** Association between a range of milk production variables in cows and (i) replacing a T allele with a C allele in the TLR4-2021 SNP (standard error in parenthesis), and (ii) TLR4-2021 genotypes

				Genotype^1^			
					
Trait	Mean Trait Value^2^	Allele Substitution (T→C)	P-value	TT	TC	CC	P-value
Milk Yield (kg)	4455 (1200)	-65.7 (137.9)	NS	0	-22.81 (226.7)	-96.04 (282.8)	NS
Protein Yield (kg)	164 (40)	-3.86 (4.56)	NS	0	-1.64 (7.48)	-6.24 (9.34)	NS
Fat Yield (kg)	197 (51)	-4.88 (5.25)	NS	0	1.24 (8.63)	-7.01 (10.77)	NS
Lactose Yield (kg)	206 (52)	-1.29 (5.83)	NS	0	-1.68 (9.55)	-1.63 (11.93)	NS
Protein Percentage^3 ^(*1000)	36 (3)	-0.39 (0.19)	< 0.05	0	0.03 (0.32)	-0.63 (0.39)	< 0.05
Fat Percentage^3 ^(*1000)	44 (8)	-0.69 (0.44)	NS	0	0.77 (0.72)	- 0.97 (0.9)	< 0.01
SCS (log_e _SCC)	1.67 (0.46)	-0.05 (0.05)	NS	0	-0.08 (0.07)	-0.11 (0.09)	NS

^1 ^Referent class is the TT genotype and is compared with the CT and CC genotype

^2 ^Mean values observed for each phenotype investigated (standard deviation in parenthesis)

^3 ^A value of 1, before multiplication, equates to 1 percentage unit

NS = Not significant

**Table 3:** The associations (standard error in parenthesis) between the TLR4-2021 genotypes and milk composition variables in cows within early, mid and late lactation

			Genotype^1^			
				
Trait	Lactation Stage (days)	Mean Trait Value^2^	TT	TC	CC	P-value
Protein Percentage^3 ^(*1000)	1 - 70	34.3 (3.3)	0	-0.15 (0.78)	1.07 (0.98)	NS
	71 - 140	35.6 (3.9)	0	0.92 (0.82)	0.09 (1.04)	NS
	141 - 305	39.3 (4.5)	0	0.23 (0.39)	-1.17 (0.51)	< 0.001
Fat Percentage^3 ^(*1000)	1 - 70	42.9 (6.4)	0	0.12 (0.32)	-0.26 (0.41)	NS
	71 - 140	41.6 (7.9)	0	-0.06 (0.35)	-0.61 (0.44)	NS
	141 - 305	47.4 (9.2)	0	1.45 (0.91)	-1.06 (1.15)	< 0.01

^1 ^Referent class is the TT genotype and is compared with the CT and CC genotype

^2 ^Mean values observed for each phenotype investigated (standard deviation in parenthesis)

^3 ^A value of 1, before multiplication, equates to 1 percentage unit

NS = Not significant

Although the C allele of TLR4-2021 was associated with decreased milk protein percentage over the entire lactation (Table 2), this association occured in late lactation (lactation days 141 to 305) (Table 3). In addition, the heterozygous TC genotype associated with an increase in milk fat percentage from days 141 to 305 of lactation (Table 3). Interestingly this association did not hold when the genotype was included in the model as a class effect (Table 2). CXCR1-777 tended to associate with SCS throughout the entire lactation when the polymorphism was included as either a class or continuous effect in the statistical model (Table 4). However, no associations were observed between CXCR1-777 and alternative mastitis indicators, *i.e*. peak SCC (highest SCC record per cow per lactation) and subclinical mastitis(SCC > 250,000 cells/ml) (data not shown).

**Table 4:** Association between a range of milk production variables in cows and (i) replacing a C allele with a G allele in the CXCR1-777 SNP (standard error in parenthesis), and (ii) CXCR1-777 genotypes

				Genotype^1^			
					
Trait	Mean Trait Value^2^	Allele Substitution (C→G)	P-value	CC	CG	GG	P-value
Milk Yield (kg)	4455 (1200)	-6.03 (101.7)	NS	0	-259.3 (192)	-66.77 (206.6)	NS
Protein Yield (kg)	164 (40)	-0.47 (3.36)	NS	0	-9.237 (6.346)	-2.81 (6.83)	NS
Fat Yield (kg)	197 (51)	-1.05 (3.87)	NS	0	-9.06 (7.31)	-3.61 (7.87)	NS
Lactose Yield (kg)	206 (52)	-0.45 (4.29)	NS	0	-13.68 (8.1)	-3.89 (8.72)	NS
Protein Percentage^3 ^(*1000)	36 (3)	0.05 (0.1)	NS	0	0.1 (0.3)	0.1 (0.3)	NS
Fat Percentage^3 ^(*1000)	44 (8)	-0.2 (0.3)	NS	0	0.7 (0.6)	-0.1 (0.6)	NS
SCS (log_e _SCC)	1.67 (0.46)	-0.06 (0.03)	< 0.1	0	0.02 (0.06)	-0.1 (0.07)	< 0.1

^1 ^Referent class is the CC genotype and is compared to the CG and GG genotype

^2 ^Mean values observed for each phenotype investigated (standard deviation in parenthesis)

^3 ^A value of 1, before multiplication, equates to 1 percentage unit

NS = Not significant

### Holstein-Friesian bulls

The genotype and allele frequencies of TLR4-2021 and CXCR1-777 in 848 Holstein-Friesian sires are listed in Table 5, with summary statistics listed in Table 6. Both SNPs deviated significantly from Hardy Weinberg equilibrium with a lower than expected frequency of TT and CC genotypes for TLR4-2021 and CXCR1-777, respectively. TLR4-2021 did not associate with any milk composition traits investigated (Table 6). The G allele of CXCR1-777 was associated with a 1.01 kg increase in fat yield (P < 0.05; Table 6).

**Table 5:** Genotype frequencies, minor allele frequencies (MAF) and significance of deviation from Hardy-Weinberg equilibrium (HWE) for the SNPs investigated within the Holstein-Friesian bull cohort

SNP^1^	Genbank	BTA	Position	Genotype	SNP Database	Genotype Frequency (%)	MAF (%)	HWE
TLR4-2021	NM_174198	8	112436581	T/T	rs8193069	0.04	0.18	0.03
				T/C		0.27		
				C/C		0.69		
CXCR1-777	U19947	2	110617059	C/C		0.01	0.34	0.00
				C/G		0.68		
				G/G		0.31		
TLR4-452	NM_174198	8	112435012	A/A	rs8193049	0.98	0.0083	0.80815
				A/C		0.02		
TLR4-1040	NM_174198	8	112435600	A/C	rs8193053	0.02	0.01	0.81
				C/C		0.98		
TLR4-1142	NM_174198	8	112435702	A/A	rs8193055	1.00	0	
TLR4-1948	NM_174198	8	112436508	A/G	rs8193066	0.02	0.0083	0.80837
				G/G		0.98		
CD14-1908	EU148609	7	51086277	A/A		0.84	0.08	0.25
				A/G		0.15		
				G/G		0.01		
CARD15-3168	AY518737	18	18169217	A/A	rs43710288	0.25	0.49	0.18698
				A/T		0.48		
				T/T		0.27		
IL8-182	AY849380	6	91792392	A/A	rs43707839	0.29	0.47	0.32
				A/G		0.48		
				G/G		0.23		
IL8-1203	AY849380	6	91793413	T/T	rs41255761	1.00	0.00	
IL8-1226	AY849380	6	91793436	A/A	rs41255762	0.29	0.49	0.69521
				A/G		0.49		
				G/G		0.22		
SERPINA1-164	X63129	21	59307330	A/A	rs41257077	0.11	0.33	0.74
				A/G		0.45		
				G/G		0.44		
SERPINA1-269	X63129	21	59307225	C/C		0.45	0.34	0.46
				C/T		0.42		
				T/T		0.13		
SERPINA1-284	X63129	21	59307210	G/G		0.44	0.35	0.18
				G/T		0.43		
				T/T		0.13		
SERPINA1-407	X63129	21	59307087	C/C	rs41257068	0.11	0.35	0.07
				C/G		0.47		
				G/G		0.42		
SERPINA1-989	X63129	21	59304656	C/C		0.43	0.34	0.94
				C/T		0.46		
				T/T		0.11		

^1 ^SNPs genotyped by iPLEX gold technology (Sequenom)

**Table 6:** Allele substitution effect (standard error in parenthesis) between SNPs and milk performance within Holstein-Friesian sires

SNP	Milk Yield	Protein Yield	Fat Yield	Protein Percent	Fat percent	SCS
	(kg)	(kg)	(kg)	(% *100)	(% *100)	(log_e _SCC *100)
Average DYD^1^	150 (237)	5.6 (6.8)	5.7 (7.9)	1.29 (7.02)	0.65 (13.96)	3.16 (11.38)
TLR4-2021 (T→C)	-14.4 (12.29)	0.41 (0.34)	-0.67 (0.44)	0.01 (0.01)	-0.01 (0.01)	0.01 (0.01)
CXCR1-777 (C→G)	2.86 (13.06)	-0.02 (0.36)	1.01 (0.46) *	0.02 (0.01)	-0.01 (0.01)	-0.01 (0.01)
CD14-1908 (A→G)	-27.43 (16.18) †	-0.98 (0.45) *	-1.32 (0.57) *	-0.01 (0.01)	-0.01 (0.01)	-0.01 (0.01)
CARD15-3168 (A→T)	-6.36 (9.57)	-0.31 (0.36)	-0.21 (0.34)	-0.01 (0.01)	0.01 (0.01)	0.01 (0.01)
IL8-182 (A→G)	8.31 (9.52)	0.32 (0.26)	0.52 (0.34)	0.01 (0.01)	0.01 (0.01)	-0.01 (0.01)
SERPINA1-164 (A→G)	-5.19 (10.23)	-0.28 (0.28)	-0.55 (0.36)	-0.29 (0.37)	-0.64 (0.77)	0.7 (0.61)
SERPINA1-269 (C→T)	13.3 (10.32)	0.48 (0.29) †	-0.74 (0.36) *	-0.04 (0.38)	-2.34 (0.77) **	0.65 (0.62)

^1 ^Average DYD values for each phenotype investigated (standard deviation in parenthesis) Significance of difference from zero ^† ^= P < 0.1; * = P < 0.05; ** = P < 0.01.

Table 5 also summaries the allele frequencies and genotypes of an additional 14 polymorphisms. Both IL8-1203 and TLR4-1142 were monomorphic with only the T and A allele present, respectively and were therefore not included in the association analysis. TLR4-452, TLR4-1040, and TLR4-1948 were also excluded from the association analyses due to the absence of the homozygous CC, AA, and AA genotypes respectively and the low proportion of the heterozygous genotypes. Minor allele frequency in the remaining SNPs ranged from 8% to 49%.

Linkage disequilibrium (LD), based on the r^2 ^statistic, between SNPs SERPINA1-164, SERPINA1-407 and SERPINA1-989 was > 0.95 (Table 7). As such, only association analysis of SERPINA1-164 was reported in Table 6. SERPINA1-269 and SERPINA1-284 also exhibited strong LD with a score of 0.99 (Table 7) with SERPINA1-284 omitted from the analysis listed in Table 6. LD between IL8-182 and IL8-1226 was 0.99 (data not shown), allowing omission of IL8-1226 from Table 6.

**Table 7:** Linkage disequilibrium (r^2^) between SNPs in coding region of *SERPINA1 *gene

SNP	SERPINA1-164	SERPINA1-269	SERPINA1-284	SERPINA1-407
SERPINA1-269	0.262			
SERPINA1-284	0.268	0.997		
SERPINA1-407	0.961	0.274	0.282	
SERPINA1-989	0.958	0.273	0.275	0.997

The associations among the seven segregating SNPs and milk production traits are listed (Table 6). The G allele of CD14-1908 was associated with decreases (P < 0.05) in milk fat and protein yield by 1.32 kg and 0.98 kg respectively and also tended to associate with decreased milk yield (P < 0.1). The T allele of SERPINA1-269 associated with 0.74 kg (P < 0.05) and 2.34% (P < 0.01) decreases in fat yield and fat percentage respectively. The T allele of SERPINA-269 also showed a tendency (P < 0.1) to associate with an increase in protein yield by 0.48 kg. The remaining SNPs, SERPINA1-164, CARD15-3168 and IL8-182, did not associate with any of the milk production traits investigated (Table 6).

Using the five SNPs in *SERPINA1*, four possible haplotypes were constructed in the Holstein-Friesian bulls (Table 8). The haplotypes G_164_T_269_T_284_G_407_C_989_, ACGCT, GCGGC were present in the population in almost equal proportions with only a 1% incidence of the GCGCT haplotype (Table 8). Bulls with the GCGGC haplotype had superior genetic merit for milk protein yield and milk fat percentage compared to the referent haplotype, GTTGC (P < 0.05), with no differences in milk yield. The ACGCT haplotype had a superior genetic merit for milk fat yield (P < 0.05) compared to GTTGC.

**Table 8:** Regression coefficients (standard errors in parenthesis) of the performance traits in the Holstein-Friesian bulls on the different haplotypes in the sequence of SERPINA-164, SERPINA1-269, SERPINA1-284, SERPINA1-407, and SERPINA1-989 in the *SERPINA1 *gene

	GTTGC^†^	GCGCT	ACGCT	GCGGC
Frequency	35%	1%	33%	31%
				
Milk yield (kg)	0	-43.1 (90.22)	-11.48 (23.71)	-38.65 (23.65)
Protein yield (kg)	0 ^a^	-2.44 ^ab ^(2.5)	1.75 ^ab ^(0.84)	1.53 ^b ^(0.84)
Fat Yield (kg)	0 ^a^	-0.47 ^ab ^(3.19)	1.75 ^b ^(0.84)	1.53 ^ab ^(0.84)
Protein percent (%*100)	0	-2 (3.31)	0.44 (0.87)	-0.22 (0.87)
Fat percent (%*100)	0 ^a^	0.93 ^ab ^(6.75)	3.95 ^a ^(1.77)	5.47 ^b ^(1.77)
SCS (log_e _SCC*100)	0	-0.04 (5.37)	-1.8 (1.42)	-1.16 (1.43)

^† ^GTTGC haplotype taken to represent the referent haplotype

^a, b, ^Regression coefficients with different superscript letters are different (P < 0.05) from each other

## Discussion

The modern dairy cow has been subjected to intense selection for milk production which has adversely affected its health [31,32]. The G allele of the CXCR1-777 SNP showed a significant association with increased milk fat yield in Holstein-Friesian bulls and a tendency to associate with decreased SCS in the on-farm study. In agreement, Leyva-Baca et al. [28] reports a tendency for the G allele to associate with increased fat yield, but not SCS, in Canadian Holstein-Friesian bulls. Furthermore, Goertz et al. [33] failed to identify an association between CXCR1-777 genotype and SCS in German Holsteins. However, Youngerman et al. [18] demonstrates an association between higher SCS and the heterozygous GC genotype compared to the GG genotype in Holstein-Friesian cows. Interestingly, Holstein-Friesian cows with high SCS have reduced daily fat production and higher lipolysis compared to low SCS herdmates [34]. In other species, CXCR1 is expressed by adipocytes [35], and its ligand, IL8, inhibits adipogenesis through down-regulation of the transcription factor peroxisome proliferator-activated receptor-γ (*PPAR-γ*) gene [36]. IL8 and CXCR1 may indirectly influence fatty acid synthesis in the mammary gland through this *PPAR-γ *regulation [37]. Alternatively, the association of *CXCR1 *with milk fat yield may simply be explained by the mapping of quantitative trait loci (QTL) for fat yield and percentage [9] at 110 to 130 Mbp on BTA2. Indeed a QTL for SCS is also linked to BTA2 at position 105 Mbp [9].

The CC genotype of TLR4-2021 was associated with a decrease in milk protein percentage in late lactation across all breeds within the on-farm study. However, this association did not persist within the Holstein-Friesian bull population. This discrepancy between the two studies may be a function of 1) differences in phenotypes and accuracy of phenotypes used (actual cow phenotypic records adjusted for systematic environmental effects or predicted transmitting abilities), 2) differences in the parity of the progeny or period during which the phenotype was measured, 3) the mean performance of the animals or the environment they were exposed to (*i.e*. genotype by environment interaction) and 4) the different genetic background and breed of the animals in the study and how that may affect interactions between the polymorphism and background genes. It is worth noting that there was a lower frequency of TLR4-2021 CT heterozygotes (0.26) in the Holstein-Friesian bull population than reported in the Holstein-Friesian cows (0.46). This discrepancy reflects the small sample size of the on-farm study.

Flow cytometry analysis demonstrates that heterozygous AG animals of CD14-1908 have higher numbers of neutrophils expressing CD14 molecules [22]. This may confer the neutrophils with altered sensitivity to LPS [22] which may aid in their fight against pathogen attack. While no association with SCS was established within our study, to the authors' knowledge this is the first report of the G allele of CD14-1908 associating with lower milk fat and protein yields and tending to associate with a decrease in milk production.

SERPINA1 levels are known to affect milk composition milk quality over the lactation curve [29]. The synonymous SERPINA1-269 polymorphism demonstrated an association with milk fat yield and milk fat percentage. Similarly, Khatib et al. [30] also found associations with a *SERPINA1 *haplotype and milk traits (milk yield, fat yield and protein yield). In our study animals, the GCGGC haplotype had similar genetic merit for milk yield but a superior genetic merit for milk protein yield and milk fat percentage when compared to GTTGC animals. Although the GTTGC haplotype associates with a decrease in SCS in a North American herd [30], it failed to associate with SCS in our bull data set. Interestingly, within our data set, the haplotype GTTGT was absent and the previously unreported GCGCT haplotype occurred in 7 bulls (1%).

## Conclusion

Of the sixteen polymorphisms in seven immune related genes genotyped, only CXCR1-777 tended to associate with SCS albeit only in the on-farm study. Interestingly polymorphisms in the immune genes, *CXCR1*, *CD14*, *SERPINA1*, did associate with milk composition. Animals with the GCGGC *SERPINA1 *haplotype associated with superior genetic merit for milk protein yield and fat percentage. The lack of an association between the polymorphisms with SCS in the Holstein-Friesian data set would question the potential importance of these variants in selection for improved mastitis resistance in the Holstein-Friesian cow.

## Methods

### DNA extraction and genotyping of cow samples

A total of 246 dairy cows (77 Holstein-Friesian, 47 Jersey, 51 Norwegian Red and 24 Montbeliarde, 47 Holstein-Friesian x Jersey crossbreeds) from a single experimental dairy farm were milked twice daily. Blood sampling, performed under license from the Department of Agriculture, Fisheries and Food by trained personnel, was taken from the tail vein by caudal venipuncture and collected into tubes containing potassium ethylenediaminetetraacetic acid (EDTA K3E 15%, 0.12 ml; BD Vacutainer, BD Vacutainer Systems, Plymouth, UK). Blood samples were initially centrifuged at 2500 × *g *for 20 min at 4°C. Buffy layers containing white blood cells were collected and DNA extracted using the QIAGEN Flexigene DNA kit (QIAGEN Ltd., West Sussex, UK), according to manufacturer's instructions. DNA was quantified and quality assessed spectrophotometrically and by gel electrophoresis.

All cows on the farm were genotyped by polymerase chain reaction-restriction fragment length polymorphism (PCR-RFLP). *CXCR1 *PCR primers were designed based on the NCBI http://www.ncbi.nlm.nih.gov nucleotide sequence U19947.(Forward: 5'-caa tac aac gaa atg gcg gat gat-3', Reverse: 5'-cag gtt gta ggg cag cca gca gag-3'). *TLR4 *primers were designed based on the sequence NM_174198 (Forward: 5'-aac agg tag ccc aga cag cat ttc-3', Reverse: 5'-ggc acg ccc tcc tcc aag tt-3'). Each PCR product was designed to incorporate a *Bsp 1286 I *restriction site within the amplicon to act as an internal quality control for enzyme activity. Cycling conditions were as follows: initial denaturation at 94°C for 5 min followed by 30 cycles for 94°C for 1 min, annealing temp (61°C for CXCR1, 60°C for TLR4) for 1 min, 72°C for 1 min, with a final extension at 72°C for 5 min. Each PCR reaction was performed in 25 μl volumes and contained 1× NH_4 _Buffer (160 nM (NH_4_)_2_SO_4_, 670 mM Tris-HCl (pH8.8), 0.1% Tween 20), 200 μM each dNTP and 2.5 U Taq Polymerase (Bioline Ltd., London, UK). *CXCR1 *PCR amplified a region of 202 bp using 100 ng template genomic DNA (gDNA), 10 pmol of each primer in the presence of 2.5 mM MgCl_2 _and 10% DMSO. *TLR4 *PCR reactions amplified a region of 580 bp using 200 ng of template gDNA, 25 pmol of each primer in the presence of 1.5 mM MgCl_2_. PCR products were analyzed by gel electrophoresis (1.5%) and ethidium bromide staining and visualized using the Alphaimager (Alpha Innotech, San Leandro, CA, USA). Restriction digests were performed in a final volume of 25 μl containing 15 μl of PCR product, 1 U of *Bsp1286 I *restriction enzyme, and 1× BSA. Restriction digests were incubated at 37°C for 3 hours, with a subsequent heat inactivation of the enzyme at 65°C for 20 mins.

Allele specific fragments of all products were visualized on a 2.5% agarose gels following electrophoresis. To confirm accuracy of genotyping by RFLP, PCR products from a subset of 20 animals were sequenced (Cogenics and MWG-Biotech). Sequence data was analyzed using Lasergene 6 (DNAStar Inc., Madison WI, USA). Control homozygous and heterozygous samples, identified by sequencing, were included in each digest as a quality control measure.

### DNA extraction and genotyping of bull samples

Semen straws from 848 Holstein-Friesian sires, representing the Irish dairy germplasm in recent years, were collected for genomic DNA extraction. Semen was washed twice in phosphate buffered saline (pH7.4) and centrifuged. The resulting cell pellets were resuspended in 450 μl of pre-warmed extraction buffer (10 mM Tris pH8, 10 mM EDTA, 0.1% SDS, 100 mM NaCl) and 15 μl of 2-mercaptoethanol was subsequently added. Following addition of 10 μl proteinase K (20 mg/ml), samples were incubated for 15 minutes at 55°C. An overnight incubation at 60°C ensured complete lysis. DNA was extracted using the Maxwell^® ^instrument (Promega Corp., Madison, WT, USA) according to the manufacturer's instructions. All SNPs were genotyped in the bull population by Sequenom^® ^using the iPLEX Gold assay on a MassARRAY^® ^platform http://www.sequenom.com. As a quality control measure, genomic DNA representing twenty-five animals was genotyped in duplicate for each SNP. Concordance across all duplicates was 100%.

### Cow phenotypic data and editing

Cows were primarily grass-fed (100% pyrennial grass) and housed during winter months. Cows were milked twice daily. Prior to milking, teats were wiped. Post milking, teats were treated with iodine. Milk yield was recorded daily. Milk sample were collected once weekly from successive morning and evening milkings and the Milkoscan 203 instrument (Foss Electric, Hillerød, Demark) was used to determine milk fat, protein and lactose concentrations. Somatic cell count was determined from milk samples using a Bentley Somacount 300^® ^(Bentley Instruments Inc., Chaska MN, USA). Subclinical mastitis events were defined as the number of SCC records greater than 250,000 cells/ml per animal per lactation. Peak SCC was the highest SCC record per animal per lactation. All animals genotyped were included in the association analysis (i.e. no selection was applied). The 246 dairy cows represented 71 sire families. As well as entire lactation totals, records were divided into 3 groups for analysis; early (days 1-70), mid (days 71-140) and late (days 141-305) lactation. Pedigree files to determine relatedness were constructed using sire information extracted from the Irish Cattle Breeding Federation (ICBF).

### Bull phenotypic data and editing

ICBF evaluated data, which included daughter yield deviations (DYDs) and predicted transmitting abilities (PTAs), as well as associated reliabilities, for a range of performance traits, was available for inclusion in the analysis. A description of models used in Irish genetic evaluations and variance components is summarized in Berry et al. [38]. The average co-ancestry among the sires was 2.2%. A repeatability model across the first five lactations is used to estimate DYD for 305-day milk, fat and protein yield and geometric mean SCS (log_e _SCC). DYDs expressed on the scale of PTA were used in this study. PTAs were de-regressed according to Berry et al. [39]. Using the method of Harris and Johnson [40], the parental contribution of the reliability of each DYD or PTA was removed. Only sires with reliability, less parental contribution, of greater than 60% were included in association analysis. For milk, fat and protein yield, as well as fat and protein percentage, a total of 742 sires were included in analysis, while 703 sires were suitable for association analysis of SCS.

### Statistical analysis

#### Cow Dataset

The association between each SNP and performance was quantified using linear mixed models in ASREML [41] with an additive genetic effect and permanent environmental effect included as random effects. Relationships among individuals were accounted for using a numerator relationship matrix. Fixed effects included in the model were contemporary group defined as experimental treatment-by-year, parity and breed. The ratio of the additive genetic to phenotypic variance and permanent environmental variance to phenotypic variance were fixed to give a heritability and repeatability of 0.35 and 0.5 respectively for milk, fat, protein, and lactose yield; 0.52 and 0.6 for fat and protein percentage and 0.11 and 0.13 for SCS, subclinical mastitis and peak SCS. SNPs were included in the model as either continuous variables or as class effects. SNPs were included in the model either individually or together. Significance was based on the F-statistic.

#### Bull Dataset

The association between each SNP and performance was quantified using weighted mixed models in ASREML [41] with bull included as a random effect, and average expected relationships among bulls accounted for through the numerator matrix. Year of birth (divided into 5 yearly intervals) and percentage Holstein of the individual bull were included as fixed effects in the model. In all instances the dependent variable was DYD for 305-day milk yield, fat yield, protein yield, and average lactation SCS, weighted by their respective reliabilities less the parental contribution. Each SNP was individually included in the model as a continuous variable. For *SERPINA1*, a multiple regression mixed model with all segregating SNPs included in the model was also undertaken. In a separate analysis, the probability of each haplotype for the bull was included as a fixed effect in the weighted animal mixed model with year of birth of the bull and Holstein proportion included as fixed effect; the most frequent haplotype (G_164_T_269_T_284_G_407_C_989_) was not included in the model to avoid linear dependencies among haplotype effects. Significance of associations was determined by the *F*-test and the appropriate degrees of freedom.

## Authors' contributions

CB, SC and DAM performed genomic DNA extractions and PCR-RFLP. CB performed statistical analysis and drafted and revised the manuscript. DB participated in the national bull study design and performed statistical analysis. LG, MD, and TMC participated in design of research, drafting and revising of manuscript. All authors read and approved the final manuscript.
